# Labour market position of young people and premature mortality in adult life: A 26-year follow-up of 569 528 Swedish 18 year-olds

**DOI:** 10.1016/j.lanepe.2021.100048

**Published:** 2021-02-11

**Authors:** Nora Döring, Michael Lundberg, Christina Dalman, Tomas Hemmingsson, Finn Rasmussen, Alma Sörberg Wallin, Susanne Wicks, Cecilia Magnusson, Anton Lager

**Affiliations:** a Department of Medical Epidemiology and Biostatistics, Karolinska Institutet, Sweden; b Department of Global Public Health, Prevention Intervention and Mechanisms in Public Health (PRIME Health), Karolinska Institutet, Sweden; c Department of Global Public Health, Epidemiology of Psychiatric Conditions, Substance Use and Social Environment (EPiCSS), Karolinska Institutet, Sweden; d Centre for Epidemiology and Community Medicine, Region Stockholm, Stockholm, Sweden; e Institute of Environmental Medicine, Karolinska Institutet, Stockholm, Sweden; f Departmet of Public Health Sciences, Stockholm University, Stockholm, Sweden; f Department of Global Public Health, Karolinska Institutet, Sweden, Retired

**Keywords:** Education, unemployment, school dropout, mortality, mental health, NEET

## Abstract

**Background:**

Throughout the industrialized world, demand for low skilled labour is falling. The length of schooling is increasing in response, but so is the proportion of individuals not finishing upper secondary school. The objective of this study was to evaluate the associations between labour market positions at age 18 and all-cause and suicide- and accident-specific mortality in later adulthood.

**Methods:**

Labour market positions at age 18 were categorized for all Swedes born 1972-77 (n=630 959) into four main groups: employed, successful students, students not about to qualify (SNAQs), and individuals not in employment, education or training (NEETs). Cox proportional hazard models were fitted to assess all-cause, suicide and accident mortality up to 2016 (ages 39-44), adjusting for high school grades, parental and own prior psychiatric diagnoses, and childhood socioeconomic status.

**Findings:**

SNAQs had substantially increased all-cause (men: HR=2.10; 95% CI 1.92-2.28, women: HR=1.64; 95% CI: 1.44-1.86), suicide (men: HR=2.16; CI: 1.86-2.51, women: HR=2.10; 95% CI 1.64-2.69), and accident specific (men: HR=2.08; 95% CI 1.77-2.44, women: 1.87; 95% CI 1.33;2.62) mortality risks compared to successful students. The risks were similar for NEETs. There was no increased risk among full-time employed compared to successful students.

**Interpretation:**

Expanding the educational system may be a natural response to falling demand for low skilled labour but not by far one that corrects the major societal challenge of it. Unless educational systems adequately respond to this challenge, only more inequality is to be expected ahead.

**Funding:**

This work was supported by a grant to FR and AL from the Swedish Research Council for Health, Working Life, and Welfare with contract number (2014-2009).

**Research in context**

**Evidence before this study**

We did a literature search both on PubMed and on Google Scholar to identify relevant scientific articles addressing the association between labour market position and in particular non-finishing education and health-related outcomes. We used the following search terms in combined or individuals searches: “High school”, “School”, “University”, “dropout/drop-out/drop out”, “Non-completion”, “NEET”, “Youth unemployment”, “Labor/Labour market”. No restriction on language, publication dates or study design were applied. Further studies were identified from the reference lists of the preliminary search results. Several relevant studies were identified, yet the majority focusing on youth unemployment. Unemployment is an important indicator of ill-health and a pressing public health concern of our times. Yet, the concept of unemployment does not sufficiently capture labour market exclusion among young people. Instead, there has been an increased focus on those “not in education, employment or training” (NEETs). This concept however does not capture those being enrolled in secondary education but not finishing it with qualification.

**Added value of this study**

Another central labour market categorization has been introduced, namely students not about to qualify (SNAQ), which is needed to capture the consequences of more inclusive educational policies. In comparison to successful students, being a student not about to qualify (SNAQ) was associated with markedly elevated risks of later premature mortality. The SNAQ estimates were as high as those for the hitherto more discussed NEETs, but the former group was about five times as large, highlighting a major societal and public health problem that presently seems overlooked. Not continuing education but instead entering the labour force with a full-time low-skilled job is not associated with an increased mortality risk, which possibly suggests the need for future policy alternatives.

**Implications of all the available evidence**

In times of more and more automation (less demand for low skilled workers), specialization (more demand for highly trained workers) and expanded educational systems - our study point to the urgent importance of closely monitoring the development for the new labour market group that SNAQ constitute.

Alt-text: Unlabelled box

## 1 Introduction

### 1.1 Background

Exclusion from the labour force is recognized as an important indicator of ill-health and a pressing public health issue of our times [1], [2], [3]. A changing labour market in which fewer, and more highly skilled, workers are needed is particularly troublesome for immigrants and young people [4]. The concept of unemployment does not capture the labour market exclusion of young people, since unemployment is often conditional on an affiliation to the job market that young people do not have. Hence, an additional labour market class, “Not in Education, Employment or Training” (NEET) [5] has been used in recent years, improving research and policy-making. Even so, an important group of young people remain overlooked in the current taxonomy, namely students who are enrolled in education, but do not complete it with a formal qualification. The current paper is focusing on this group described as “Student(s) Not About to Qualify” (SNAQ).We are especially interested in this group of students who would have, most likely in earlier times, not continued schooling but instead would have entered the labour market with low-skilled jobs that are no longer available. We hypothesize that being exposed to an educational environment that is not suitable might have negative (mental) health consequences which go beyond the negative health consequences resulting from a lack of a qualifying degree, referred to as “scarring effect” [6].

It is important to make SNAQs visible in a policy context since expansion of the educational system has been the main political response to the changing labour market situation. Expansion is designed not only to qualify the highly skilled workers needed on an increasingly demanding labour market, but also to keep youth exclusion from the labour market at bay when there is a lack of low-skilled job opportunities. When Sweden went through a deep economic recession in the early 1990s [7], a lot of mainly low-skilled jobs were quickly lost, especially for young people [8]. Since most young people <20 years are not yet established on the labour market, this job loss did not mainly translate into increased unemployment, but rather into increased enrolment in upper-secondary school. Upper-secondary education is formally voluntary in Sweden, and, up to the late 1980s, around 20% instead entered the labour force after finishing compulsory school at the age of 16. Ever since the economic crisis in the 1990s, however, nearly 100% enter upper-secondary education [9].

In the early 1990s, the Swedish school system was still considered among the best in international comparisons [10]. Yet, enrolling virtually complete birth cohorts, including students who had failed in compulsory school, has been a major challenge to secondary schools. As a result, a sizeable proportion of students since the 1990s have not obtained formal qualifications for either work or further studies. As in many other Western countries, the proportion of students failing to complete secondary school in Sweden is now around 20-30% [9, 11].Despite a growing body of research on the adverse health effects of youth unemployment, little is known, however, about the health-related consequences of increasing enrolment in upper-secondary school and the resulting increase in the number of SNAQs.

Sweden of the 1990s offers us an opportunity to study the long-term consequences of exposure to a complete spectrum of labour-market positions, that is: having earnings from work corresponding to a full-time (low skill) employment, having earnings lower than full-time but higher than the absolute poverty level, being a student about to qualify, being a NEET, or being a SNAQ (the group we have just introduced). We are in a position to investigate six consecutive total birth cohorts, while controlling for mental health status (own and parental), high school grades, and childhood socioeconomic position. Here, we examine how labour market position at age 18 is associated with all-cause mortality and mortality from suicides and accidents up to middle age.

## 2 Methods

### 2.1 Study population

**Fig. 1:**
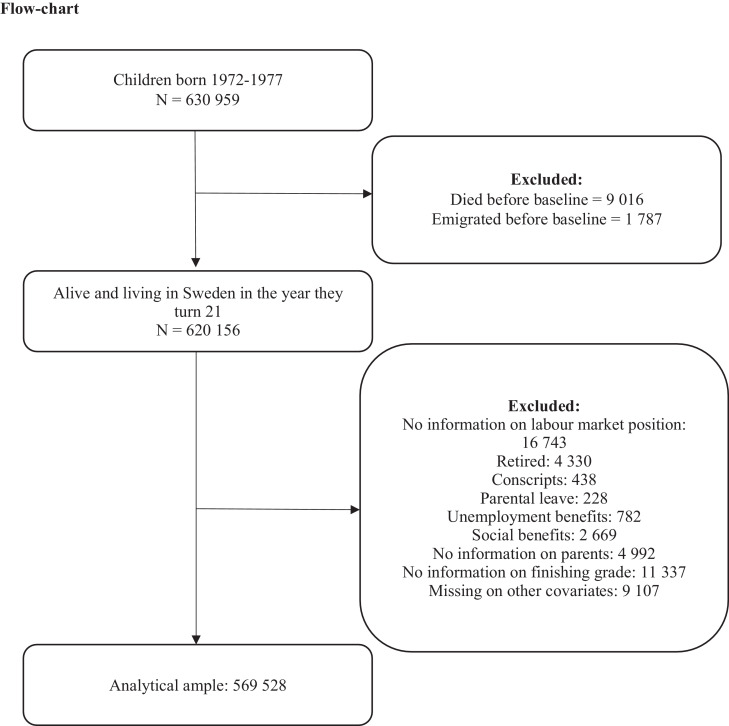
Flowchart of the analytical sample.

The study population is based on the Swedish Total Population Register and includes virtually all individuals born in Sweden between 1972 and 1977 (n=630 959), followed-up until 2016. Individuals who died (n= 9 016) or emigrated (n= 1 787) before January of the year they turned 21 were excluded (Fig. 1).

### 2.2 Ethical approval

The study was approved by the Stockholm Regional Ethical Review Board (*(DNR 2010/1185-31/5)*).

### 2.3 Exposure variables

Labour market position during the year an individual turned 18 was categorized in four distinct categories on the basis of information from the Swedish Longitudinal Integration Database for Health Insurance and Labour Market Studies (LISA) and the Swedish Register on Education (UREG). Student status was defined by receipt of student benefits and by completing secondary studies before or during the year the individual turned 20. SNAQs were defined as persons who received student benefits the year they turned 18, but who did not complete secondary studies (as evidenced by not having a finishing grade recorded) before or during the year they turned 20. In Sweden, all enrolled students receive automatically student benefits. Employment was defined as having a work-based yearly income that exceeded the recommended norm for a reasonable living standard for each year that a cohort member turned 18 (1990 to 1995). To reduce the risk of reverse causality we excluded those on long-term sick-leave or in early retirement (n=4 330). Furthermore, we excluded those with unemployment benefits (n=782), the conscripted (n=438) and people on parental leave (n=228) due to small numbers (Figure 1).

### 2.4 Outcomes

We assessed all-cause mortality and mortality due to suicides (ICD-9 codes E950-959, E980-E989; ICD-10 codes X60-X84, Y10-Y34) and accidents (ICD-9 codes E800-E949, E960-E999; ICD 10 codes V01-X59) using data from the Cause of Death Register. To avoid underestimation and consistent with previous studies, injures of undetermined intent were included in the suicide category [12].

### 2.5 Covariates

The covariates included were: parental country of birth (Sweden; outside of Sweden), parental socio economic status at child's birth (high non-manual; low non-manual; skilled manual; unskilled manual) [13, 14], parents’ highest obtained education at child's birth (university education; upper-secondary education; compulsory education (9 years of education); less than 9 years of education) own primary school grades at age 15 (0-1.9; 2.0-2.9; 3.0-3.9, 4.0-5.0, where 5 is the highest possible grade), own and parental hospitalization with a psychiatric diagnosis (ICD F00-F99) (yes/no) before baseline, and year of birth (dummy) to capture the role of finishing compulsory school during the economic recession in the early 1990’s. Evidence from earlier studies has shown that these variables are associated with both labour market position at age 18 and mortality outcomes in midlife and were evaluated in our cohort for their association with the exposure (Table 1) and outcomes (eTable 1).

**Table 1:** Description of the analytical sample.

	Employed	Student	SNAQ	NEET
	N (%)	N (%)	N (%)	N (%)
Total=569 528	30 444	477 543	50 991	10 550
Sex (male)	18 171 (59.7)	244 739 (51.3)	24 617 (48.3)	3 830 (36.3)
Year of birth (median)	1973 (1972-1974)	1974 (1973-1976)	1975 (1973-1976)	1974 (1973-1975)
School grade				
0.0-1.9	4 485 (14.7)	8 173 (1.7)	8 268 (16.2)	2 819 (26.7)
2.0-2.9	14 435 (47.4)	125 424 (26.3)	22 911 (44.9)	4 634 (43.9)
3.0-3.9	9 930 (32.6)	268 114 (56.1)	17 177 (33.7)	2 334 (22.1)
4.0-5.0	1 594 (5.2)	75 832 (15.8)	2 635 (5.2)	763 (7.2)
Mother not born in Sweden	2 895 (9.4)	38 245 (8.0)	5 866 (11.5)	1 541 (14.6)
Father not born in Sweden	3 222 (8.6)	40 895 (8.6)	6 695 (13.1)	1 762 (16.7)
Parental SES				
Non-manual (high)	8 892 (29.2)	230 480 (48.3)	17 863 (35.0)	3 132 (29.7)
Non-manual (low)	5 511 (18.1)	77 000 (16.1)	7 767 (15.2)	1 405 (13.3)
Skilled manual	8 615 (28.3)	102 923 (21.6)	12 648 (24.8)	2 774 (26.3)
Unskilled manual	7 426 (24.4)	67 140 (14.0)	12 713 (24.9)	3 239 (30.7)
Psychiatric diagnosis	975 (3.2)	7 254 (1.5)	3 725 (7.3)	875 (8.3)
Psychiatric diagnosis (mother)	2 167 (7.1)	22 315 (4.7)	4 753 (9.3)	1 059 (10.0)
Psychiatric diagnosis (father)	2 717 (8.9)	25 478 (5.3)	5 616 (11.1)	1 236 (11.7)

Notes: SNAQ= Student not about to qualify; NEET= Not in Education, Employment or Training

### 2.6 Statistical analyses

The associations between labour market position in young adulthood and all-cause, suicide-, and accident-specific mortality were investigated in crude and multivariable Cox proportional hazards regression models with attained age as underlying time scale. Person time was calculated from January 1^st^ of the year the participants turned 21 years until date of emigration, death or December 31^st^, 2016, whichever came first. The proportional hazard assumption was verified graphically by plotting log(-log(survival)) versus log of survival time, and statistically by means of Schoenfeld residuals.

Due to an indication of interaction with sex (p-value < 0.001), stratified analyses were run; and results are presented separately for males and females. In sensitivity analyses, we excluded all cohort individuals who had an inpatient mental-health related diagnosis (ICD F00-F99) before baseline. In supplementary analyses, we explored whether the associations between labour market positions and all-cause mortality differed by type of employment and split the group into employment with lower salary as proxy for a temporary employment or an employment in part-time and employment with higher salary as proxy for a full-time employment. Temporary or part-time employment was defined as having less than 2/3 of the median income based on national salary statistics for the respective year. All analyses were run in Stata 15.0.

### 2.7 Role of the funding source

This work was supported by a grant from the Swedish Research Council for Health, Working Life, and Welfare with contract number 2014-2019. The funder had no influence on study design, data collection, data analysis, interpretation, or writing of the report.

## 3 Results

The analytical sample comprised the 569 528 (91.8%) men and women with complete information on all covariates who were classified as employed, students, SNAQs or NEETs. Individuals who were excluded from the analytical sample had a higher proportion of foreign born parents and came from a lower socio-economic background compared to those individuals included in the sample. The finishing grades from compulsory school were lower among individuals who were excluded (eTable 2). At the end of follow-up, there had been 11 685 005 person-years of observation and 5 827 deaths from any cause.

The individuals classified as students had higher grades from compulsory school, and their parents a higher level of education and higher SES, than the other groups. Prior psychiatric diagnoses were over four times more prevalent among SNAQs than students; also, parental mental diagnoses were more often registered among the SNAQs and the NEETs than among the students (Table 1).The difference in median birth year between the four labour market groups suggests that there have been changes over time in labour market positions among 18 year-olds. We therefore examined the distribution of group classification across years of birth. Among both men and women there was a decline over time in the proportion of 18 year-olds who were classified as employed, and an increase in the proportion of SNAQs as well as of students. Among 18 year-old men changes in labour market positions between 1990 and 1995 showed a drop in the proportion of the cohort classified as employed from 34.6% to 2.8%, while the proportion of students increased from 60.1% to 75.1% and the proportion of SNAQs from 5.3% to 22.1%. Between 1990 and 1995 the proportion of women in the cohort classified as employed fell from 24.9% to 1.4%, while the proportion of students increased from 67.0% to 78.1%; SNAQs increased in proportion from 8.1% to 20.5%. A test for trend was significant for both men (z=170.0, p<0.001) and women (z=127.0, p<0.001).

The associations between labour market position at age 18 and mortality in later adulthood are shown in Tables 2, 3 and 4. In Model 1 (adjusted for birth year) SNAQs at age 18 had a statistically significant more than threefold increased risk of dying prematurely compared to peers who left secondary school with a qualification. The estimates were attenuated by 50%–70% in the adjusted models, yet remained statistically significant for all-cause mortality, suicide-specific mortality, and accident-specific mortality. The associations for NEETs were similar, yet somewhat less pronounced. There was a slightly increased risk for the employed compared to students. In supplementary analyses (eTable 3), where the employment group was split, there was no statistically significant differences between the full-time (low-skilled) employed and students graduating with qualification in the adjusted model. Generally, the associations between unfavourable labour market position and mortality were more pronounced in men than in women. However, for females, being employed at age 18 was associated with an almost threefold increased risk in suicide mortality compared to being a student, whereas no statistically significant association was discerned in men.

**Table 2:** Associations between labour market position at age 18 and all-cause mortality of Swedes born 1972-77; HR (95% CI).

	Cases/n	Model 1	Model 2	Model 3
**Men**		HR (95% CI)	HR (95% CI)	HR (95% CI)
Employed	365/18 171	1.72 (1.54;1.93)	1.24 (1.10;1.38)	1.21 (1.07;1.35)
Student	2 615/244 739	*Ref.*	*Ref.*	*Ref.*
SNAQ	825/24 617	3.27 (3.02;3.54)	2.34 (2.15;2.55)	2.10 (1.92;2.28)
NEET	164/3 830	4.08 (3.48;4.78)	2.43 (2.06;2.86)	2.12 (1.80;2.50)
**Women**				
Employment	122/12 737	1.62 (1.34;1.95)	1.33 (1.09;1.61)	1.25 (1.03;1.52)
Student	1 297/232 804	*Ref.*	*Ref.*	*Ref.*
SNAQ	345/26 374	2.37 (2.10;2.67)	1.92 (1.69;2.18)	1.64 (1.44;1.86)
NEET	94/6 720	2.40 (1.95;2.97)	1.78 (1.42;2.22)	1.54 (1.23;1.92)

Notes: SNAQ= Student not about to qualify; NEET= Not in Education, Employment or Training; Model 1: Adjusted for year of birth; Model 2: Additionally adjusted for parental country of birth (Sweden; outside of Sweden), parental SES (high non-manual; low non-manual; skilled manual; unskilled manual), parents’ highest obtained education (university education; upper-secondary education; compulsory education; less than compulsory education), primary school grades (0-1.9; 2.0-2.9; 3.0-3.9, 4.0-5.0); Model 3: Additionally adjusted for own and parental prior inpatient psychiatric diagnosis and prior inpatient self-harm diagnose.

**Table 3:** Associations between labour market position at age 18 and suicide-specific mortality of Swedes born 1972-77; HR (95% CI).

	Cases/n	Model 1	Model 2	Model 3
**Men**		HR (95% CI)	HR (95% CI)	HR (95% CI)
Employed	104/18 171	1.68 (1.36;2.07)	1.20 (0.97;1.49)	1.15 (0.93;1.43)
Student	791/244 739	*Ref.*	*Ref.*	*Ref.*
SNAQ	286/24 617	3.70 (3.23;4.24)	2.57 (2.24;2.90)	2.16 (1.86;2.51)
NEET	52/3 830	4.28 (3.23;5.66)	2.44 (1.82;3.27)	1.97 (1.46;2.65)
**Women**				
Employed	45/12 737	3.10 (2.23;4.29)	2.69 (1.93;3.76)	2.32 (1.66;3.26)
Student	275/232 804	*Ref.*	*Ref.*	*Ref.*
SNAQ	108/26 374	3.43 (2.75;4.30)	2.93 (1.78;4.08)	2.10 (1.64;2.69)
NEET	27/6 720	3.38 (2.27;5.02)	2.70 (1.78;4.10)	1.96 (1.28;2.99)

Notes: SNAQ= Student not about to qualify; NEET= Not in Education, Employment or Training Model 1: Adjusted for year of birth; Model 2: Additionally adjusted for parental country of birth (Sweden; outside of Sweden), parental SES (high non-manual; low non-manual; skilled manual; unskilled manual), parents’ highest obtained education (university education; upper-secondary education; compulsory education; less than compulsory education), primary school grades (0-1.9; 2.0-2.9; 3.0-3.9, 4.0-5.0); Model 3: Additionally adjusted for own and parental prior inpatient psychiatric diagnosis and prior inpatient self-harm diagnose.

**Table 4:** Associations between labour market position at age 18 and accident-specific mortality of Swedes born 1972-77; HR (95% CI).

	Cases/n	Model 1	Model 2	Model 3
**Men**		HR (95% CI)	HR (95% CI)	HR (95% CI)
Employed	109/18 171	1.96 (1.60;2.42)	1.30 (1.05;1.61)	1.28 (1.03;1.58)
Student	719/244 739	*Ref.*	*Ref.*	*Ref.*
SNAQ	244/24 617	3.43 (2.97;3.96)	2.28 (1.94;2.67)	2.08 (1.77;2.44)
NEET	49/3 830	4.46 (2.96;3.97)	2.42 (1.79;3.29)	2.16 (1.60;2.94)
**Women**				
Employed	14/12 737	1.54 (0.88;2.69)	1.25 (0.71;2.20)	1.18 (0.67;2.08)
Student	168/232 804	*Ref.*	*Ref.*	*Ref.*
SNAQ	53/26 374	2.78 (2.04;3.79)	2.19 (1.57;3.05)	1.87 (1.33;2.62)
NEET	13/6 720	2.57 (1.46;4.53)	1.86 (1.02;3.37)	1.61 (0.89;2.93)

Notes: SNAQ= Student not about to qualify; NEET= Not in Education, Employment or Training Model 1: Adjusted for year of birth; Model 2: Additionally adjusted for parental country of birth (Sweden; outside of Sweden), parental SES (high non-manual; low non-manual; skilled manual; unskilled manual), parents’ highest obtained education (University education; upper-secondary education; compulsory education; less than compulsory education), primary school grades (0-1.9; 2.0-2.9; 3.0-3.9, 4.0-5.0); Model 3: Additionally adjusted for own and parental prior inpatient psychiatric diagnosis and prior inpatient self-harm diagnose.

## 4 Discussion

### 4.1 Main findings

In a cohort of Swedish males and females born between 1972 and 1977, being a student not about to qualify (SNAQ) in upper-secondary school at age 18 was a strong risk factor for premature mortality from age 21 up to middle age from any cause, suicides or accidents in comparison to being a successful student. After adjusting for confounding variables, the effect sizes attenuated substantially, but remained with a two-fold increase in risk. The slightly increased risk related to being employed compared to being a student finishing with a qualification was largely explained by confounding, with the exception of a marked and persistent elevated risk for suicide among women.

### 4.2 Strengths and weaknesses

Major strengths of the study are that it is population-based, with a low proportion of missing values, and with 16 to 21 years of follow-up. In addition, through register linkage we were able to control for a variety of possible confounding factors. Whereas our analyses is based on nearly complete birth cohorts from 1970-1978 and followed up to 2016, it is to mention that during that period Sweden had a large amount of migration that is not captured in our sample and therefore possibly hinder generalizability to the entire Swedish population.

Health selection may explain some of the associations observed, since poor health, especially poor mental health, leads to poor school performance or an inability successfully to complete secondary school [15]. Alongside the changes on the labour market over the last decades, there has been an increase in mental health problems. Mental ill-health now accounts for 30% of the total burden of disease among young adults in Europe, and is a known risk factor for mortality outcomes in midlife [12]. Further, it has been suggested that this increase in mental health problems is closely related to poor academic achievement and labour market opportunities [13], [14], [15], [16]. We have attempted to control for selection by excluding all individuals who received benefits for early retirement or long-term sick leave (sickness benefits for at least half a year). Further, in sensitivity analyses, we excluded people with a psychiatric diagnosis before entering the study, but this did not affect the estimates. Yet, since only severe inpatient diagnoses are registered, there is a risk of misclassification, and therefore a possible overestimation of the association of labour market position with mortality, given that only around one in ten of persons receiving mental-health-related care are registered in inpatient care [16].

Our classification of employment status was fairly inclusive, that is, having an income from work beyond the bare minimum. Thus, we were unable to differentiate between secure and precarious employment, the latter being quite common in this age group and a possible determinant of ill-health [17]. A possible protective effect of employment at age 18, vis-à-vis being a SNAQ or NEET, might be even stronger for those in secure employment. In supplementary analyses, we have tried to take this into account by splitting the employed group into temporary/part-time employment and full-time employment. Indeed, full-time employment was not associated with an increased mortality risk. For women, even the all-cause analysis is based on a small number of exposed cases and these results should be interpreted with caution. Furthermore, we cannot exclude the possibility of misclassification, since our categorization is based on yearly income only, which can potentially bias our estimates in either direction.

The use of mortality as a main outcome has the advantage of having an objective and well-documented endpoint. Yet, it seems likely that the burden of disease stemming from non-completion of school is underestimated as a result, since less severe health events associated with unfavourable school-to-work transitions will not be captured.

The findings are in line with previous literature showing that higher educational attainment is protective in relation to premature mortality [18, 19]. Yet, the general policy recommendation cannot simply be more education, since it seems that the individuals who continue in secondary education but are not able to complete it with a qualification are worse off than those who did not even start secondary education but instead had (low-skilled) employment at age 18. A study from the UK, utilizing the compulsory school reform of 1972 in a regression-discontinuity design, showed, like our study, that “forcing low achieving to remain in an academic environment may have long-term unintended consequences on their mental health” [20].

Previous research has shown that unemployment is associated with higher mortality [21], and we have now found that there are similar, if not worse, increased risks for those who go on to further study but fail to obtain a secondary-school qualification – a far more common exposure in time and persons. Being a SNAQ seems as risky as being a NEET, which should be a great cause of concern given that the former group is about five times larger than the latter and was until now not visible. In addition, much of the literature has focused on labour market status among 20-25 year-olds. But studying young adults’ labour market position as early as at age 18 is important, not only to identify unfavourable trajectories and possibly intervene earlier, but also since this is a time when many young people make the transition from school to work; and those who do not go on to pursue a tertiary education are faced with entering the workforce.

The interaction effect of sex on the association of labour market position with mortality might be explained by the availability of low-skilled gender-stereotypical jobs, or by the gender-specific perceived or actual societal stigma surrounding certain labour market positions. At the same time, women tend to seek earlier and more (mental-health-related) health care (23), which might be protective in terms of later mortality and explain why the overall risk of an unfavourable labour market position is more pronounced in men. Yet, these explanations are only speculative.

With almost one-third of the student population finishing secondary education without a qualification, it is fair to say that not completing secondary school is not an individual problem but a public issue requiring a resolution that goes beyond efforts at individual level. Thus, impelling young adults to continue to study may be actively harmful to a substantial proportion of them if there are insufficient resources to support individuals in completing their studies and obtaining a qualification. Given that at least part of the association between employment position and mortality is explained by prior (unobserved) mental ill-health, adolescents with mental health problems need to be better supported and treated. With the concurrent increases in mental health problems and the number of SNAQs, more research is needed to disentangle the two trends and shed light on the direction of association between them.

### 4.3 Conclusion

Being enrolled but not completing upper-secondary school with a qualification was found to be associated with substantially increased long-term risks of all-cause and of cause-specific mortality. The question whether non-completion simply indicates the start of an unfavourable labour market trajectory, or whether there is possibly also a variable with an independent effect on premature mortality, remains to be answered. Yet, at times when about one-third of students are not completing school in time, and one fifth may never, effective responses at societal level seem urgently needed.

## Declaration of interests

The authors declare that they have nothing to disclose.

## Appendix Supplementary materials

Image, application 1
