# Supplementary material for: Labour market position of young people and premature mortality in adult life: A 26-year follow-up of 569 528 Swedish 18 year-olds
Source: Lancet Reg Health Eur. 2021 Feb 11;3:100048. doi: 10.1016/j.lanepe.2021.100048 (PMC8454531; doi:10.1016/j.lanepe.2021.100048)
Supplement: Supplementary file 1 [file mmc1.docx]

**Supplementary material**

**eTable 1 Bivariate association between the outcomes and covariates, HR (95% CI)**

|  | **All-cause mortality** | **Suicide** | **Accident** |
| --- | --- | --- | --- |
|  | N = 5 827 | N = 1 688 | N = 1 369 |
| **Sex (male)** | 2·02 (1·91;2·13) | 2·56 (2·30;2·85) | 4·27 (3·72;4·90) |
| **Year of birth** | 1·01 (1·00;1·03) | 1·04 (1·01;1·07) | 1·02 (0·99;1·06) |
| **School grade** |  |  |  |
| **0·0-1·9** | 6·26 (5·57;7·05) | 6·29 (5·07;7·82) | 12·71 (9·50;17·00) |
| **2·0-2·9** | 2·82 (2·54;3·13) | 2·70 (2·22;3·26) | 5·13 (3·91;6·73) |
| **3·0-3·9** | 1·38 (1·24;1·53) | 1·26 (1·04;1·53) | 2·12 (1·61;2·79) |
| **4·0-5·0** | *Ref* | *Ref* | *Ref* |
| **Mother not born in Sweden** | 1·46 (1·35;1·59) | 1·84 (1·60;2·10) | 1·32 (1·11;1·57) |
| **Father not born in Sweden** | 1·46 (1·35;1·58) | 1·85 (1·62;2·11) | 1·44 (1·23;1·70) |
| **Parental SES** |  |  |  |
| **Non-Manual (high)** | *Ref* | *Ref* | *Ref* |
| **Non-manual (low)** | 1·08 (0·99;1·17) | 1·03 (0·89;1·20) | 1·21 (1·03;1·43) |
| **Skilled manual** | 1·33 (1·24;1·42) | 1·21 (1·07;1·37) | 1·50 (1·31;1·72) |
| **Unskilled manual** | 1·59 (1·49;1·71) | 1·78 (1·57;2·01) | 1·84 (1·59;2·12) |
| **Parental Education** |  |  |  |
| **University** | *Ref* | *Ref* | *Ref* |
| **Upper secondary** | 1·23 (1·16;1·31) | 1·18 (1·05;1·32) | 1·44 (1·25;1·64) |
| **Compulsory** | 1·32 (1·21;1·43) | 1·09 (0·93;1·32) | 1·68 (1·42;2·00) |
| **< Compulsory** | 1·35 (1·24;1·47) | 1·16 (0·98;1·337) | 1·66 (1·38;2·00) |
| **Psychiatric diagnosis** | 4·59 (4·21;5·02) | 7·59 (6·63;8·69) | 4·37 (3·63;5·26) |
| **Self-harm diagnosis** | 4·82 (4·19;5·54) | 9·42 (7·79;11·41) | 3·14 (2·21;4·46) |
| **Psychiatric diagnosis (mother)** | 1·87 (1·71;2·03) | 2·82 (2·46;3·24) | 1·92 (1·61;2·30) |
| **Psychiatric diagnosis (father)** | 1·80 (1·65;1·95) | 2·14 (1·85;2·47) | 1·92 (1·63;2·27) |

**eTable 2 Comparison of individuals included in the final analytical sample to those excluded from the analytical sample**

|  | **Excluded from the cohort** | **Included in the cohort** |
| --- | --- | --- |
|  | (n= 56 428) | (n= 569 528) |
| **Year of birth (median)** | 1974 (1973;1976) | 1974 (1973;1976) |
| **Male** | 33 289 (54·2) | 291 357 (51·2) |
| **Mother born outside of Sweden** | 19 973 (33·1) | 48 527 (8·5) |
| **Missing** | 1 078 |  |
| **Father born outside of Sweden** | 19 593 (36·5) | 52 574 (9·2) |
| **Missing** | 7 709 |  |
| **School grade** |  |  |
| **0·0-1·9** | 3 343 (15·8) | 23 745 (4·2) |
| **2·0-2·9** | 8 139 (38·4) | 167 404 (29·4) |
| **3·0-3·9** | 7 784 (36·7) | 297 555 (52·3) |
| **4·0-5·0** | 1 930 (9·1) | 80 824 (14·2) |
| **Missing** | 40 235 |  |
| **Parental SES** |  |  |
| **Non-manual (high)** | 12 494 (29·0) | 260 367 (45·7) |
| **Non-manual (low)** | 4 947 (11·5) | 91 683 (16·1) |
| **Skilled manual** | 7 422 (17·2) | 126 960 (22·3) |
| **Unskilled manual** | 18 281 (42·4) | 90 518 (15·9) |
| **Missing** | 18 281 |  |
| **Maternal psychiatric diagnosis** | 4 942 (8·0) | 30 294 (5·3) |
| **Paternal psychiatric diagnosis** | 4 699 (7·7) | 35 047 (6·2) |

**eTable 3 Associations between labour market position at age 18 and all-cause mortality in Swedes born 1972-77; HR (95% CI)**

|  | **Cases (n)** | **Model 1** | **Model 2** | **Model 3** |
| --- | --- | --- | --- | --- |
| **Men** |  |  |  |  |
| Part-time/ temporary employment | 290 | 1·80 (1·59;2·04) | 1·33 (1·17;1·51) | 1·29 (1·13;1·46) |
| Full-time employment | 75 | 1·48 (1·18;1·87) | 0·99 (0·78;1·26) | 0·98 (0·77;1·23) |
| Student | 2 615 | *Ref.* | *Ref.* | *Ref.* |
| SNAQ | 825 | 3·27 (3·02;3·54) | 2·34 (2·15;2·55) | 2·09 (1·92;2·28) |
| NEET | 164 | 4·08 (3·48;4·77) | 2·45 (2·08;2·89) | 2·12 (1·79;2·50) |
| **Women** |  |  |  |  |
| Part-time/ temporary employment | 109 | 1·61 (1·32;1·96) | 1·36 (1·11;1·67) | 1·27 (1·04;1·56) |
| Full-time employment | 13 | 1·68 (0·97;2·91) | 1·31 (0·76;2·29) | 1·25 (0·72;2·17) |
| Student | 1 297 | *Ref.* | *Ref.* | *Ref.* |
| SNAQ | 345 | 2·37 (2·10;2·67) | 1·92 (1·69;2·18) | 1·65 (1·44;1·87) |
| NEET | 94 | 2·40 (1·95;2·97) | 1·78 (1·43;2·22) | 1·53 (1·22;1·91) |

Notes: SNAQ= Student not about to qualify; NEET= Not in Education, Employment or Training Model 1: Adjusted for year of birth; Model 2: Additionally adjusted for parental country of birth (Sweden; outside of Sweden), parental SES (high non-manual; low non-manual; skilled manual; unskilled manual), parents’ highest obtained education (University education; upper-secondary education; compulsory education; less than compulsory education), primary school grades (0-1·9; 2·0-2·9; 3·0-3·9, 4·0-5·0); Model 3: Additionally adjusted for own and parental prior inpatient psychiatric diagnosis and prior inpatient self-harm diagnose
